# Comparative evaluation of corn, coconut, and coconut testa oils in sodium alginate-stabilized nanoemulsions processed by microfluidization

**DOI:** 10.1038/s41598-026-49545-5

**Published:** 2026-04-26

**Authors:** Hewa Pathiranage Dilani Thilanka Hewa Pathirana, Anna Zimoch-Korzycka, Dominika Kulig, Anna Maria Krawczyk, Shima Vahedi

**Affiliations:** Department of Functional Food Product Development, Faculty of Biotechnology and Food Science, Wroclaw University of Environmental and Life Sciences, Chelmonskiego 37, Wroclaw, 51-630 Poland

**Keywords:** Coconut oil, Corn oil, Microfluidization, Nanoemulsion, Sodium alginate, Testa oil, Tween 80, Biotechnology, Engineering, Materials science, Nanoscience and technology

## Abstract

The limited stability of conventional emulsions has led to the development of sodium alginate-stabilised nanoemulsions, which offer enhanced functionality for food applications. This study aimed to formulate nanoemulsion stabilized by sodium alginate (0.5% w/v) and Tween 80 (1% v/v) using three types of oil (1% v/v): corn, coconut, and testa oil. The particle size of the emulsion was initially reduced by homogenizing a coarse emulsion (15000 rpm/ 5 min), followed by five passes through the microfluidizer. Changes in the physicochemical properties of the emulsions during microfluidization and over 24 days of storage were evaluated. The pH (5.2–6.2), and electrical conductivity (1090 − 1160 µS/cm) of the emulsions varied depending on the number of microfluidic passes, oil type, and storage duration. The third microfluidization pass produced significantly smaller nanoparticles (111.75 nm) with enhanced stability, exhibiting no signs of flocculation or creaming. Emulsions prepared with testa and coconut oils showed, lower zeta potential (–9.93 mV), higher whiteness index (59.547), viscosity (0.003859 Pa·s), and thixotropy of hysteresis loop area (9.742 Pa/s) compared to those prepared with corn oil. The emulsion containing testa oil and processed through the third microfluidization pass demonstrated the most favourable characteristics throughout the storage period. Overall, both the number of microfluidization passes and the type of oil significantly influenced the behaviour of sodium alginate-stabilized nanoemulsions.

**Supplementary Information:**

The online version contains supplementary material available at 10.1038/s41598-026-49545-5.

## Introduction

Targeted delivery of bioactive compounds has gained increasing attention due to its potential health benefits in preventing chronic diseases. An emulsion is a colloidal delivery system in which water and oil phases are combined using low- molecular-weight surfactants that reduce the interfacial tension between the two phases. In oil-in-water emulsions, the hydrophobic tail of the surfactant adsorbs onto oil droplets, while the hydrophilic polyoxyethylene head interacts with the aqueous phase^1^. Thus, coalescence between oil droplets is prevented by the surfactant barrier. However, conventional emulsions are often less stable and can be affected by factors such as temperature and pH. Over time, they may undergo destabilization process, including creaming, flocculation, coalescence, and Ostwald ripening during storage^2^.

The properties of an emulsion can be influenced by the type of oil in the minor phase. Corn oil is rich in polyunsaturated fatty acids, particularly linoleic acid (C18:2), which constitutes approximately 50%-60% of its composition. Moreover, the presence of phytosterols and tocopherols in Corn oil contributes to its oxidative stability by acting as antioxidants^3^. Coconut oil is a saturated fat containing a high proportion (45%-50%) of medium-chain fatty acids, particularly lauric acid (C12:0). These medium-chain triglycerides contribute to its distinctive melting characteristics, oxidative stability, and antimicrobial properties (Dayrit, 2020). Coconut testa is the brown outer layer of coconut fruit, and a byproduct of coconut processing industries. The testa oil contains a blend of unsaturated and saturated fatty acids, along with phenolic and other natural antioxidant compounds^4^. These compositional differences can affect the physicochemical properties, functional properties and behaviours of the emulsion.

Moreover, emulsions are further enhanced using various strategies to prevent phase separation. Studies have shown that a three-dimensional alginate network can form an internal structure within the colloidal system, thereby improving emulsion stability^5^. Additionally, alginates in colloidal systems facilitate direct food applications, such as edible coatings, films, and capsules. Sodium alginate, a hydrophilic biopolymer extracted from brown seaweed, is an environmentally friendly polysaccharide widely used in food applications. It is an anionic natural salt composed of linear chains of D-mannuronic acid (M-block) and L-guluronic acid (G-block), linked by β (1→4) glycosidic bonds^6^. These monomer units can be arranged in homopolymeric (e.g., MMM, GGG) or heteropolymeric (e.g., MGMG) sequences^7^.

The anionic nature of sodium alginate imparts a negative charge to the aqueous phase through its carboxylate (COO⁻) groups. This, in turn, induces a negative charge on the hydrophilic head groups of non-ionic surfactants, promoting electrostatic repulsion between adjacent oil droplets^8^. Previous studies have demonstrated that macromolecules can stabilize oil droplets through a combination of steric hindrance and electrostatic repulsion^9^. Furthermore, the ionic gelation ability of the sodium alginate chains enables formation of an ‘egg-box’ structure through specific intermolecular interactions^10^. However, the length of the linear polymer chains and their sequential arrangement determine the physicochemical properties of sodium alginate solutions^11^. To modify these properties, high-energy mechanical methods such as high-pressure homogenization, ultrasonication, and microfluidization can be employed.

Among various techniques, microfluidization has gained considerable attention due to its effectiveness in reducing particle size to the nanoscale. This method combines two mechanisms: water jet technique and high-pressure homogenization. The high shear stress generated during microfluidization increases the surface area of molecules within the emulsion and contributes to the shortening of sodium alginate chains. As a result, multiple microfluidization passes can significantly influence the structural behaviour of the sodium alginate network surrounding oil droplets. Additionally, the type and structure of fatty acid chains in the oil phase, along with the behaviour of non-ionic surfactants, play a critical role in the formation of a stable oil–water interface within the colloidal system^12^.

Particle size reduction is crucial for developing stable emulsions with desirable functional properties. Moreover, nano-sized emulsions offer several advantages over conventional emulsions. Nanoemulsions with droplet sizes below 200 nm provide a significantly larger surface area, which facilitates the rapid release and enhanced absorption of bioactive compounds during digestion^13^. The increased surface area of oil nanodroplets enables the adsorption of higher concentrations of surfactants and carboxylate (COO⁻) groups, thereby enhancing electrostatic repulsion. These properties make nanoemulsions highly suitable for the controlled release of bioactive compounds in drug delivery and functional food applications^8^. Moreover, the presence of a polysaccharide matrix surrounding lipid droplets increases system sensitivity and enables the selective release of encapsulated compounds under specific pH and thermal conditions^14^. Despite these benefits, few studies have investigated the combined use of conventional emulsifiers and polysaccharide-based networks to develop modified emulsions.

Moreover, most previous studies have concentrated on single-oil systems, whereas comparative studies involving oils with different fatty acid profiles, such as corn, coconut, and coconut testa oils, are limited. Differences in saturation, polarity, and interfacial behaviour among these oils may influence emulsion formation, droplet size distribution, and stability during storage. Therefore, it is important to understand the interaction between these oils and a sodium alginate-surfactant system under high-pressure homogenization conditions in designing stable colloidal systems. Moreover, limited attention has been given to systems in which a layer of sodium alginate forms a dense network stabilized on top of the polyoxyethylene head groups of non-ionic surfactants. Therefore, this study aimed to investigate the effect of microfluidization passes on the behaviour of the sodium alginate network in Tween 80-stabilized nanoemulsions formulated with corn, coconut, and testa oils, and to assess the stability of these emulsions over 24 days of storage.

## Methodology

### Materials and chemicals

Sodium alginate (A1112, low viscosity, brown algae, Lot # SLCG0203, Sigma-Aldrich, Burlington, Massachusetts, USA), Tween 80 (HLB = 15, Sigma-Aldrich, Burlington, Massachusetts, USA) and ultra-pure water (Pol-water system of DL3-150 with osmosis membrane, Poland, Warsaw) with specific electrical conductivity of 0.055 µS/cm were used. It is equipped with a sediment filtration unit, an adsorption filtration unit, a reverse osmosis unit, a double iron exchange unit and a UV disinfection unit (254 nm) to produce water for laboratory purposes to fulfil PN-EN ISO 3696:1999 and PN-EN 60746-3:2006 laboratory standards.

The coconut oil (BIOFOOD™) and corn oil (BASSO) were purchased from the local market in Wroclaw, Poland. The coconut testa oil was imported from the Coconut Research Institute in Sri Lanka. The peroxide values of the oils were: Coconut oil (Not detected) (Dayrit, 2020), Coconut testa oil (0.90 ± 0.31 meq O_2_/kg)^4^, Corn oil (1.58 ± 0.45 meq O_2_/kg)^3^.

### Preparation of coarse emulsion and nanoemulsion

A sodium alginate solution (0.5% w/v) was mixed with 1% (v/v overall) Tween 80 and 1% (v/v) of each type of oil (corn/ coconut/ testa oil). The mixture was pre-homogenized using a high-shear homogenizer (IKA@T25 Ultra TURRAX, Germany; Rod 18G S25 EC C/220002409 G – coarse rotor-stator geometry) at 15,000 rpm for 5 min to produce a coarse emulsion without cooling. Subsequently, the coarse emulsion was processed through microfluidizer (Microfluidics M-110P, Netherlands, Interaction chamber F12Y diamond Y-type (75 μm)), using a 200 MPa pressure range without any back-pressure to reduce the particle size to the nanoscale. During the microfluidization, the temperature of the sample was maintained at 8 °C ± 3 °C.

Each formulation underwent five consecutive microfluidization passes. The emulsions were stored under refrigerated conditions (4 ± 2 °C) to simulate typical storage conditions for emulsion-based food products and to minimize microbial and oxidative changes that could affect the physicochemical stability of the system during the evaluation period (0 days to 24 days).

### Quality evaluation of nanoemulsion

#### pH value and electrical conductivity (EC)

The pH and electrical conductivity of the nanoemulsions were measured using a Seven Multi™ model S40 meter (Mettler Toledo, Warsaw, Poland). Measurements were conducted at 25 ± 2 °C using an InLab^®^ Routine Pro pH probe and an InLab^®^ 731 conductivity probe (Mettler Toledo).

#### Particle size and zeta potential

The average droplet size (Z-average diameter), polydispersity index (PDI) (droplet diameter of a mixture of sodium alginate, tween 80 and oil) and zeta potential of the nanoemulsions were determined using dynamic light scattering (DLS) with a Zetasizer PRO analyzer (Malvern Instruments Ltd., UK). The samples were used without dilution and filtration at 25 °C. The viscosity is used as 0.887 cP. The Non-Invasive Back Scatter (NIBS) technique with a 173° measurement angle was used for sample detection.

#### Whiteness index

The color parameters of the emulsions were assessed using a CIE digital chroma meter (MINOLTA CR-400) equipped with a CR-A33d light projection tube (ø 22 mm disc; Konica Minolta, Osaka, Japan). The device was calibrated with a standard white calibration plate (CR-A33a; Konica Minolta, Osaka, Japan) with the reference values: Y = 93.8, x = 0.3133, and y = 0.3195. The L* (lightness), a* (red–green), and b* (yellow–blue) values were recorded, and the Whiteness Index (WI) was calculated using the following equation (Eq. 1), as described by Acevedo-Fani^8^.

1 $$\:WI=100-\sqrt{{\left(100-{L}^{*}\right)}^{2}+{(a}^{*2}+{b}^{*2}})$$

#### Creaming index

The height of the cream layer (HI) in the nanoemulsion was measured at three time points: initially (day 0), after 12 days, and after 24 days, along with the total height of the nanoemulsion (HE). The Creaming Index (CI) was calculated according to Eq. 2, as described by Wangpradit et al.^15^.

2 $${\text{Creaming Index }}\left( {{\mathrm{CI}}} \right)\% ~ \frac{Height \; of \; the \; cream \; layer\:\left(HC\right)}{Height \: of \: the \: emulsion \: \left(HE\right)}\:\times\:100\%$$

#### Rheological properties of emulsion

The rheological properties of the experimental nanoemulsions were determined using a HAAKE MARS 60 rheometer (Thermo Scientific, Karlsruhe, Germany) at a temperature of 20 °C. All tests were performed using a cone probe (C35/1° Ti – 02230302) and a measuring plate (TMP35, stainless steel 18/8) in controlled rate (CR) mode with a specific range of shear rate. The measurements were managed using RheoWin Job Manager software (Haake).

##### Flow property

Shear stress (σ) and viscosity (η) values were recorded over an increasing shear rate range of 600–7760 s⁻¹ within 2 min. Prior to measurement, each sample was stabilized for 2 min at a constant shear rate of 5 s⁻¹ at 20 °C. The experimental data were further fitted using the Herschel–Bulkley model (Eq. 3) and the Ostwald–de Waele model (Eq. 4) as follows:

3 $$\tau = \tau _{0} + k \times \gamma ^{{ \cdot n}}$$

4 $$\tau = k \times \gamma ^{{ \cdot n}}$$

The τ is the shear stress (Pa), K is the consistency index (Pa·s), γ̇ is the shear rate (s^− 1^), n is the flow behaviour index (dimensionless), and τ_0_ is the yield stress (Pa). The goodness of fit (R^2^) for Herschel–Bulkley model and the Ostwald–de Waele model were at 0.999.

The selected shear rate range (600–7760 s⁻¹) was applied to simulate the high mechanical stresses typically encountered during industrial processing operations (e.g., pumping, mixing, and homogenization) and to evaluate the flow behaviour of the emulsions under conditions relevant to practical applications.

##### Thixotropic properties

The flow behaviour with structural rigidity and recovering properties of the emulsion was analyzed using ramped-up and ramped-down flow curves over time. The hysteresis loop was obtained by increasing the shear rate from 0 s^− 1^ to 100 s^− 1^ for 2 min, followed by a maximum shear rate of 100 s^− 1^ for 2 min, and decreasing shear rate from 100 s^− 1^ to 0 s^− 1^ in 2 min. The thixotropic area of the hysteresis loop was calculated using RheoWin Data Manager software version 4.00 (Thermo Scientific, Karlsruhe, Germany).

##### Viscoelastic properties

Viscoelastic properties were analyzed using an oscillatory shear test. In the first step, the linear viscoelastic region (LVR) was determined by applying a stress sweep in the range of 0.001–10 Pa at a constant frequency of 1 Hz. Based on this, a frequency sweep was performed in the range of 0.01–10 Hz at a constant deformation amplitude of 0.01 Pa. The storage modulus (G′), loss modulus (G″), and loss tangent (tan δ) were recorded as functions of frequency. For statistical analysis, data obtained at a frequency of 1 Hz were used.

### Statistical analysis

A three-factor experimental design was used to evaluate the effect of oil type (O), microfluidization passes (M), and storage time (T) on the analyzed responses and the following model was used:

Y_ijkl_ = µ + O_i_+M_j_+T_k_+(OM)_ij_+(OT)_ik_+(MT)_jk_+(OMT)_ijk_+_εijkl_,

where Y_ijkl_ is the observed response, µ is the overall mean, O_i_ is the fixed effect of the i^th^ type of oil, M_j_ is the fixed effect of the j^th^ microfluidic passes and T_k_ is the fixed effect of the k^th^ storage time. The two-way interaction effects are represented by (OM)_ij_, (OT)_ik_, and (MT)_jk_ while the three-way interaction of the factors is represented by (OMT)_ijk_. The εijkl denotes random experimental error of the l^th^ independent replicate.

Measurements were conducted on independent samples at storage days 0, 12, and 24 The samples taken at each storage time were independently analyzed to avoid the disturbance effect (specifically for creaming index). Shapiro-Wilk and Levene tests were used to validate the assumption of ANOVA (Table 1).

**Fig. 1:**
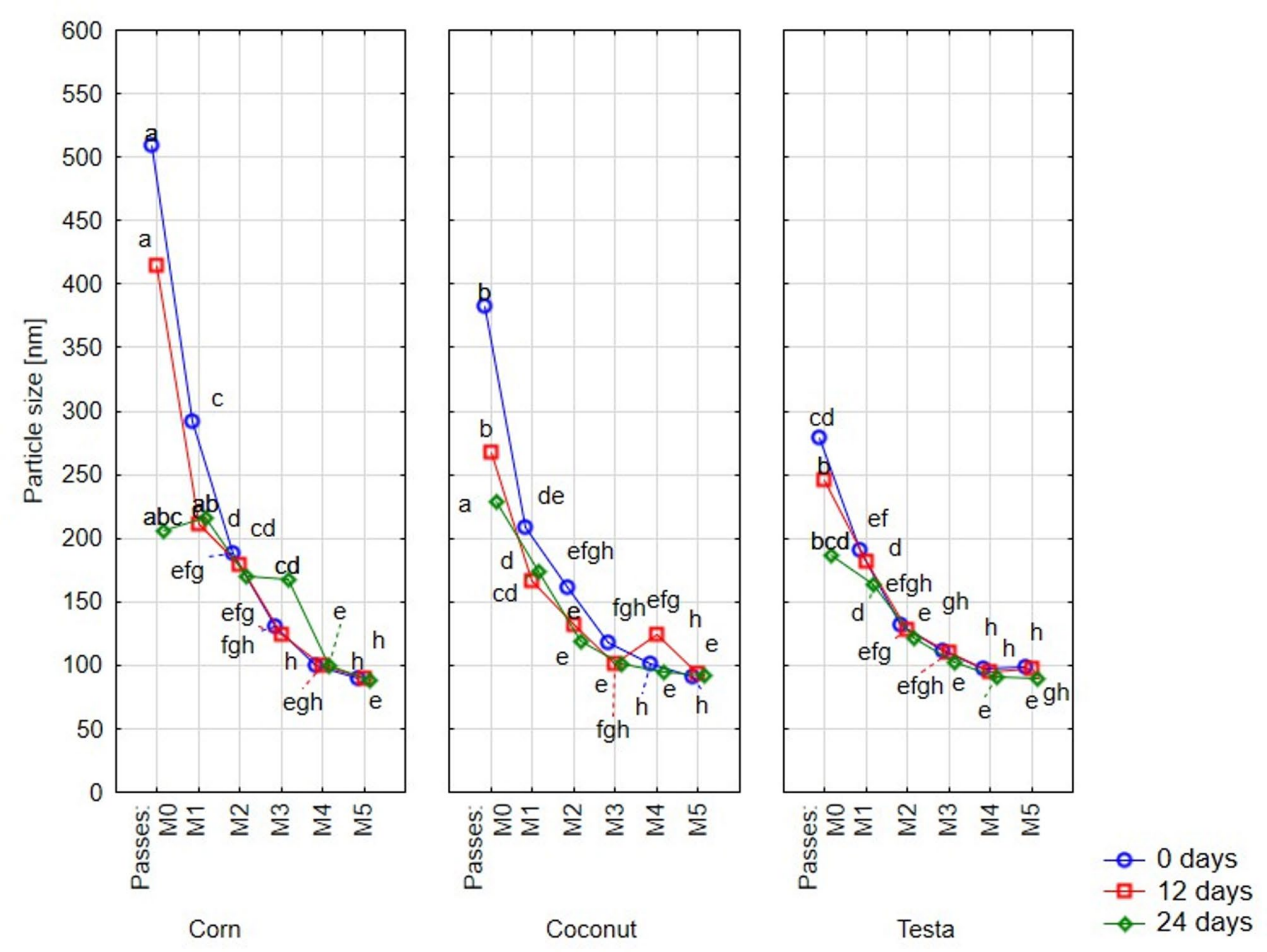
Influence of three-way interactional effect of all factors (oil type, storage time, microfluidic passes) on particle size of emulsion. Separate post hoc comparisons for each level of the time factor ( 0, 12 days, and 24 days) were presented. Within each time category, different letters indicate statistically significant differences (*p* < 0.05).

**Fig. 2:**
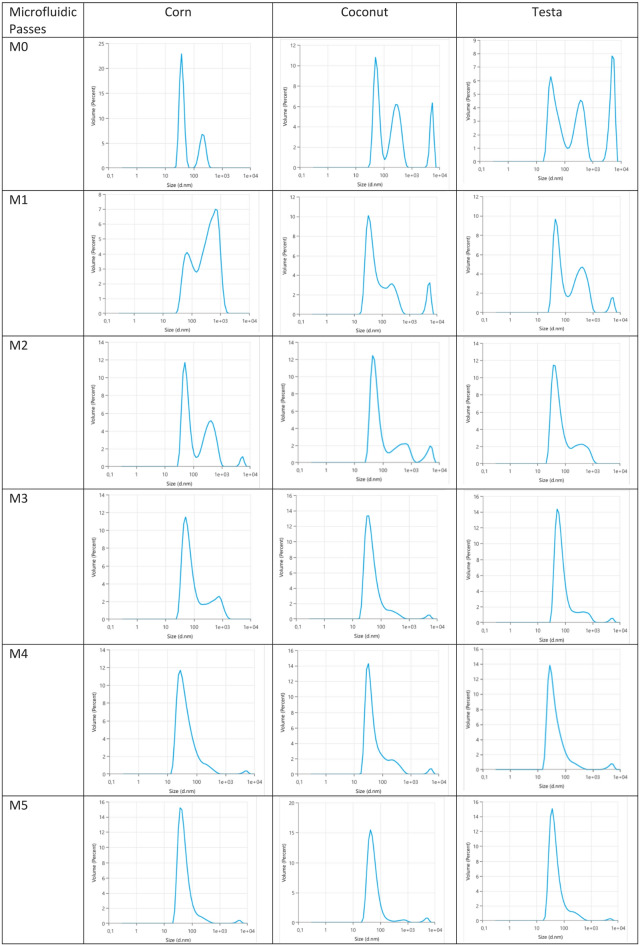
Changes in intensity vs. volume distribution of particle size of emulsions prepared with three oil types and different microfluidic passes in initial storage day.

**Fig. 3:**
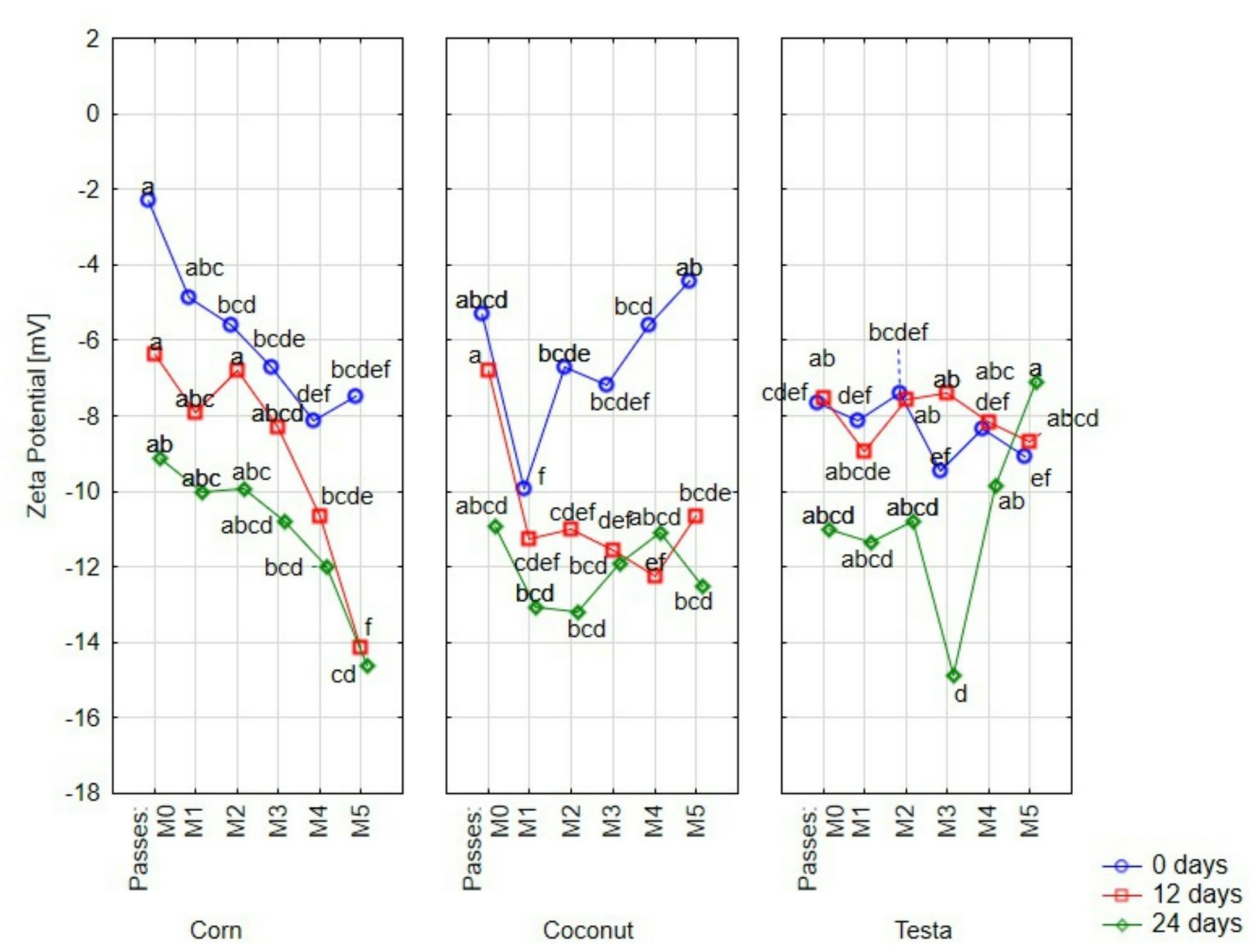
Changes in zeta potential values (interaction effect) of emulsions prepared with three oil types and microfluidic passes over the storage period. Separate post hoc comparisons for each level of the time factor (0, 12 days, and 24 days) were presented. Within each time category, different letters indicate statistically significant differences (*p* < 0.05).

The main effectoil type (O), microfluidic passes (M), and storage time (T) was analyzed and shown in Tables 2 and 3. The three-way interaction between oil type x microfluidization passes x storage time was evaluated, and it was presented as Figs. 1 and 2, and Fig. 3. Tukey test (*p* < 0.05) was used to conduct post hoc comparisons. The analyzed results of three-way interaction (oil type × microfluidic passes × storage time) were presented as Supplementary material Table S1. Principal component analysis (PCA) was also conducted to identify the major contributing variables among the measured parameters. The Statistica software (Cloud Software Group, Inc., 2023; Data Science Workbench, version 14, http://tibco.com) was used with a significance threshold set at *p* < 0.05.

**Table 1:** Variable factors.

Oil type	Microfluidic passes	Storage time [days]
Corn	M0	T0
	M1	
Coconut	M2	T12
	M3	
Testa	M4	T24
	M5	

**Table 2:** Influence of main effects: oil type, microfluidic passes, and storage time on pH, EC, particle size, zeta potential and whiteness value of emulsions.

Emulsion properties	Main effects					
	Oil type		Microfluidic passes		Storage time [days]	
pH	Corn	5.82 ± 0.18^a^	M0	5.71 ± 0.25^ab^	T0	5.61 ± 0.16^b^
			M1	5.76 ± 0.39^a^		
	Coconut	5.84 ± 0.22^a^	M2	5.69 ± 0.21^ab^	T12	5.76 ± 0.33^a^
			M3	5.69 ± 0.23^ab^		
	Testa	5.44 ± 0.17^b^	M4	5.67 ± 0.22^b^	T24	5.73 ± 0.26^a^
			M5	5.67 ± 0.27^b^		
EC [µS/cm]	Corn	1119.69 ± 11.49^b^	M0	1125.06 ± 15.36^ab^	T0	1120.60 ± 13.00^b^
			M1	1129.94 ± 16.66^a^		
	Coconut	1130.75 ± 16.39^a^	M2	1128.83 ± 14.73^a^	T12	1123.22 ± 15.59^b^
			M3	1118.83 ± 14.34^c^		
	Testa	1121.22 ± 15.63^b^	M4	1117.89 ± 16.10^c^	T24	1127.89 ± 16.67^a^
			M5	1122.78 ± 11.48^bc^		
Particle size [nm]	Corn	187.54 ± 115.68^a^	M0	302.26 ± 109.15^a^	T0	182.22 ± 116.38^a^
			M1	200.45 ± 42.75^b^		
	Coconut	153.13 ± 78.12^b^	M2	147.85 ± 27.00^c^	T12	158.92 ± 82.22^b^
			M3	118.64 ± 25.75^d^		
	Testa	140.11 ± 55.02^c^	M4	100.18 ± 11.78^e^	T24	139.64 ± 50.04^c^
			M5	92.18 ± 6.48^e^		
Polydispersity Index (PDI)	Corn	0.422 ± 0.15^a^	M0	0.571 ± 0.17^a^	T0	0.452 ± 0.16^a^
			M1	0.436 ± 0.07^b^		
	Coconut	0.419 ± 0.10^a^	M2	0.421 ± 0.03^bc^	T12	0.402 ± 0.09^b^
			M3	0.387 ± 0.03^c^		
	Testa	0.396 ± 0.11^b^	M4	0.347 ± 0.07^d^	T24	0.383 ± 0.08^b^
			M5	0.313 ± 0.07^d^		
Zeta potential [mV]	Corn	-8.65 ± 3.24^a^	M0	-7.44 ± 2.70^a^	T0	-6.90 ± 2.17^a^
			M1	-9.49 ± 2.57^bc^		
	Coconut	-9.74 ± 3.09^b^	M2	-8.77 ± 2.51^b^	T12	-9.22 ± 2.45^b^
			M3	-9.79 ± 3.29^c^		
	Testa	-9.07 ± 2.46^b^	M4	-9.56 ± 2.27^bc^	T24	-11.34 ± 2.46^c^
			M5	-9.86 ± 3.69^c^		
Whiteness index	Corn	43.84 ± 6.15^b^	M0	43.77 ± 5.11^d^	T0	43.36 ± 9.00^c^
			M1	47.36b ± 7.42^c^		
	Coconut	43.80 ± 8.39^b^	M2	46.58 ± 8.47^c^	T12	46.48 ± 7.91^b^
			M3	49.23 ± 9.60^ab^		
	Testa	55.90 ± 6.32^a^	M4	50.73 ± 10.61^a^	T24	53.70 ± 6.79^a^
			M5	49.41 ± 10.54^a^		

Different letters (a−d) in different columns for each parameters show the significant differences (*p*<0.05) among treatments due to three separate post hoc comparisons.

**Table 3:** Main effects of oil type, microfluidic passes, and storage time on rheological properties of emulsions.

Emulsion properties	Main effects					
	Oil type		Microfluidization passes		Storage time [days]	
Apparent Viscosity at 5 s-1[Pa.s]	Corn	0.0108 ± 0.0073^b^	M0	0.0213 ± 0.0104^b^	T0	0.0167 ± 0.0096^a^
			M1	0.0380 ± 0.0738a		
	Coconut	0.0229 ± 0.0154^a^	M2	0.0178 ± 0.012b	T12	0.0221 ± 0.0160^a^
			M3	0.017 ± 0.0121b		
	Testa	0.0302 ± 0.053^a^	M4	0.0150 ± 0.0117b	T24	0.0252 ± 0.0535^a^
			M5	0.0189 ± 0.0167b		
Apparent Viscosity at 7760 s-1[Pa.s]	Corn	0.0030 ± 0.0003^b^	M0	0.0035 ± 0.0004^a^	T0	0.0029 ± 0.0006^a^
			M1	0.0031 ± 0.0006^b^		
	Coconut	0.0029 ± 0.0006^b^	M2	0.0029 ± 0.0004^b^	T12	0.0031 ± 0.0004^a^
			M3	0.0030 ± 0.0003^b^		
	Testa	0.0032 ± 0.0006^a^	M4	0.0028 ± 0.0006^b^	T24	0.0030 ± 0.0005^a^
			M5	0.0028 ± 0.0004^b^		
Shear stress at 5s^− 1^ [Pa]	Corn	0.054 ± 0.037^b^	M0	0.106 ± 0.052^b^	T0	0.083 ± 0.048^a^
			M1	0.190 ± 0.369^a^		
	Coconut	0.115 ± 0.077^a^	M2	0.089 ± 0.060^b^	T12	0.110 ± 0.080^a^
			M3	0.086 ± 0.058^b^		
	Testa	0.151 ± 0.263^a^	M4	0.074 ± 0.054^b^	T24	0.126 ± 0.268^a^
			M5	0.094 ± 0.083^b^		
Shear stress at 7760 s-1 [Pa]	Corn	22.884 ± 2.266^b^	M0	26.750 ± 2.960^a^	T0	22.789 ± 4.478^a^
			M1	23.693 ± 4.635^b^		
	Coconut	22.160 ± 4.364^b^	M2	22.698 ± 3.342^b^	T12	23.668 ± 3.076^a^
			M3	22.861 ± 2.429^b^		
	Testa	24.909 ± 4.274^a^	M4	21.882 ± 4.575^b^	T24	23.495 ± 4.077^a^
			M5	22.020 ± 3.084^b^		
Flow Behavior Index n (-) Ostwald De Waele model	Corn	0.9328 ± 0.0292^b^	M0	0.9413 ± 0.0240^b^	T0	0.9412 ± 0.0318^b^
			M1	0.9394 ± 0.0309^b^		
	Coconut	0.9355 ± 0.0274^b^	M2	0.9370 ± 0.0369^b^	T12	0.9428 ± 0.0323^b^
			M3	0.9588 ± 0.0326^a^		
	Testa	0.9750 ± 0.0219^a^	M4	0.9577 ± 0.0311^a^	T24	0.9592 ± 0.0310^a^
			M5	0.9524 ± 0.0333^a^		
Consistency Index k [Pa.s]Ostwald De Waele model	Corn	0.0060 ± 0.0015^a^	M0	0.0062 ± 0.0011^a^	T0	0.0053 ± 0.0013^a^
			M1	0.0059 ± 0.0015^ab^		
	Coconut	0.0055 ± 0.0011^b^	M2	0.0055 ± 0.0013^b^	T12	0.0055 ± 0.0012^a^
			M3	0.0047 ± 0.0012^cd^		
	Testa	0.0042 ± 0.0009^c^	M4	0.0043 ± 0.0008^d^	T24	0.0049 ± 0.0015^b^
			M5	0.0047 ± 0.0013^c^		
Yield Stress [Pa] Herschel-Bulkley model	Corn	0.055 ± 0.042^b^	M0	0.052 ± 0.024^b^	T0	0.032 ± ^c^0.037
			M1	0.108 ± 0.152^a^		
	Coconut	0.050 ± 0.050^b^	M2	0.048 ± 0.045^b^	T12	0.094 ± 0.033^a^
			M3	0.055 ± 0.041^b^		
	Testa	0.087 ± 0.109^a^	M4	0.054 ± 0.040^b^	T24	0.067 ± 0.112^b^
			M5	0.068 ± 0.052^b^		
Thixotropy area [Pa/s]	Corn	4.57 ± 1.41^c^	M0	6.23 ± 2.45^abc^	T0	5.98 ± 2.51^a^
			M1	5.36 ± 1.99^c^		
	Coconut	6.24 ± 2.66^b^	M2	6.71 ± 2.89^a^	T12	5.84 ± 2.70^a^
			M3	5.52 ± 2.07^bc^		
	Testa	7.10 ± 1.76^a^	M4	5.50 ± 1.85^bc^	T24	6.09 ± 1.37^a^
			M5	6.47 ± 1.94^ab^		
Loss tangent (tan δ)	Corn	0.664 ± 0.0692^a^	M0	0.649 ± 0.145^a^	T0	0.600 ± 0.160^a^
			M1	0.655 ± 0.111^a^		
	Coconut	0.605 ± 0.167^a^	M2	0.573 ± 0.119 ^a^	T12	0.620 ± 0.160^a^
			M3	0.652 ± 0.153^a^		
	Testa	0.603 ± 0.160^a^	M4	0.578 ± 0.164^a^	T24	0.652 ± 0.133^a^
			M5	0.638 ± 0.193^a^		

Different letters (a−d) in different columns for each parameters show the significant differences (*p*<0.05) among treatments due to three separate post hoc comparisons.

## Results and discussion

### pH

The pH value of an emulsion is a key indicator of its physicochemical, microbiological, and sensory stability. The three main factors; oil type, microfluidization passes, and storage time significantly influenced the pH variation of the emulsions (Table 2). A significantly lower pH value was observed in emulsions formulated with testa oil compared to those containing coconut and corn oils. This difference can be attributed to variations in the fatty acid composition among the oil types. Corn oil is rich in long-chain unsaturated fatty acids^16^, coconut oil primarily contains medium-chain saturated fatty acids^17^, whereas testa oil exhibits a mixed profile with medium-chain saturated and a high proportion of unsaturated fatty acids^18^. The significantly (*p* < 0.05) lower pH in testa oil emulsions may be attributed to accelerated lipid oxidation of unsaturated fatty acids, as previously reported by Narayanankutty et al., 2018. Additionally, testa oil contains higher levels of polyphenolic acids than the other oils^19^, which may contribute to the increased acidity of the external aqueous phase in the emulsion.

In addition to oil type, microfluidic passes also played a significant role in pH reduction. One hypothesis is that more microfluidic passes can be linked to the gradual depolymerization of sodium alginate and a simultaneous decrease in oil droplet size. Preliminary studies further confirmed that the number of microfluidic passes changed the particle size of the sodium alginate 0.5% solution from 534.7 ± 10.22 nm to 375.8 ± 8.34 with five passes of microfluidization. This process may be associated with a reduction in the length of alginate chains and an augmentation in the quantity of terminal carboxyl (COO -) ions and with the loss of the protons (H +) and thus the acidification of the systems. Also, mechanical breakdown of fatty acids into smaller nanoparticles could be associated with the increased oxidative vulnerability that might also be contributing to the observed decrease in pH^20^. Therefore, the combined effects of oil composition, microfluidic processing intensity, and oxidative sensitivity of the system may explain the observed pH decline in nanoemulsions, particularly those containing testa oil and subjected to multiple microfluidic passes.

During storage, the pH values of the emulsions continued to fluctuate, likely due to reaggregation of the sodium alginate network^5^, neutralization of acidic compounds, hydrolysis of fatty acids, or potential microbial activity producing both acidic and alkaline metabolites. Each oil type exhibited a distinct pH trend over the 24-day storage period, although all emulsions remained within a relatively stable pH range of 5.1 to 6.2 as shown in the interaction effect of the Supplementary Table S1.

### Electrical conductivity

Electrical conductivity (EC) reflects the ionic strength of the aqueous phase of the emulsion and can be used to determine the type of emulsion formed^21^. In this study, the relatively high EC values (ranging from 1000 to 1170 µS/cm) confirmed that the system was an oil-in-water (O/W) emulsion^22^. The high EC values may be attributed to the presence of high free ions in the continuous water phase, arising from the dissociation of H^+^ of carboxyl groups and the addition of Na^+^ ions of sodium alginate.

Oil type significantly affected the electrical conductivity (EC) of the emulsions. Emulsions prepared with coconut oil exhibited higher EC values compared to those containing corn or testa oils. This may be attributed to the specific fatty acid composition of coconut oil, which is rich in medium-chain saturated fatty acids, such as lauric acid. Shorter and less interactive fatty acid chains may reduce ion trapping at the oil–water interface. This may result in a higher fraction of free ions (e.g., Na^+^, H^+^) in continuous aqueous phase, contributing to the observed rise of conductivity^20^. In contrast, corn and testa oils contain higher proportions of long-chain and polyunsaturated fatty acids, which may create more disordered or reactive interfacial environments (Dayrit, 2020),^4,3^. Such interfaces could promote greater adsorption or immobilization of ions, particularly those released from sodium alginate (COO⁻, Na⁺), thus reducing their availability in the water phase and lowering EC values. Therefore, the observed differences in electrical conductivity among oil types are likely related to their distinct fatty acid profiles and their influence on interfacial structure and ionic mobility.

In addition to oil type, microfluidic passes had a significant effect on the electrical conductivity (EC) of the emulsions. After the second microfluidic cycle, EC increased, likely due to the enhanced breakdown of sodium alginate chains into smaller fragments, resulting in proton (H⁺) release and an increased concentration of free ions in the aqueous phase^12^. However, further microfluidic processing (passes 3 to 5) led to a gradual decrease in EC values. This reduction may be attributed to the formation of a denser nanonetwork structure composed of sodium alginate. Such a structure could restrict the mobility of ions in the water phase, despite the presence of a higher number of terminal carboxyl groups (COO⁻)^23^.

The electrical conductivity (EC) of the emulsions increased significantly (*p* < 0.05) over the storage period (Table 2). This increase may be attributed to the gradual degradation of the sodium alginate network, which facilitates the release and mobility of ions in the aqueous phase^24^. In addition, oxidative degradation of fatty acids during storage may lead to the formation of low-molecular-weight acidic compounds, contributing further to the ionic content of the system^25^.

Previous studies have reported considerable changes in EC during storage, depending on emulsion composition and temperature conditions. For example,^26^ observed EC fluctuations of approximately 600 µS/cm in emulsions containing sweet al.mond and olive oil under varying storage temperatures. In contrast, the EC fluctuations observed in the present study were relatively mild, approximately 60 µS/cm, indicating higher ionic stability of the formulated nanoemulsions under the tested storage conditions.

### Particle size

Particle size is a key parameter influencing the physical stability and functional performance of emulsions. However, achieving significantly low particle size is challenging due to the internal Laplace pressure within the droplets^27^. The disruption process of droplets depends on the duration of the applied force and the flow regime of the system (laminar, elongational, or turbulent), and it should exceed the Laplace pressure^28^. The type and concentration of emulsifier have a significant (*p* < 0.05) impact on the droplet deformation process and the resulting particle size. Furthermore, the presence of alginates influences emulsification by increasing the viscosity of the continuous phase and potentially entrapping droplets within a polymeric network, which can alter droplet dynamics and limit coalescence^29^.

All three main factors, oil type, microfluidic passes, and storage time significantly (*p* < 0.05) influenced the reduction of particle size in the nanoemulsions. Among the tested oils, emulsions containing testa oil exhibited significantly smaller particle sizes compared to those formulated with corn oil and coconut oil (Table 2). The three-way interactions (oil type × microfluidic passes × storage time) are shown in Fig. 1 and Supplementary Table S1. The molecular structure of testa oil is less compact than that of coconut oil, which may facilitate more effective droplet disruption under shear forces during microfluidization, resulting in smaller particle sizes^18^. In contrast, the fatty acid profile of corn oil offers greater resistance to deformation and shear-induced breakup, making it more difficult to achieve droplet size reduction during processing. Similar findings were reported by Ahmed et al.^30^; who studied the effect of fatty acid chain length on droplet size in curcumin-loaded nanoemulsions. Their results showed that emulsions with medium-chain triglycerides produced significantly smaller droplets (174 nm) than those with long- or short-chain triglycerides, consistent with the present study. Moreover, previous research has shown a positive correlation between the concentration of sodium alginate and droplet size, indicating that higher alginate concentrations may hinder efficient size reduction due to increased viscosity and stronger interfacial barriers^11^. This highlights the importance of balancing Alginate concentration with emulsification conditions to achieve optimal droplet size in nanoemulsion systems.

Microfluidic passes effectively reduced the size of the emulsion (together with oil droplets and sodium alginate). However, no significant further reduction in particle size was observed beyond the fourth microfluidization pass. This plateau effect may be attributed to the saturation of emulsifier molecules at the droplet interface, which stabilizes the interfacial tension and maintains internal Laplace pressure that cannot be overcome by additional shear forces. As a result, optimizing both the type and concentration of emulsifiers is critical for achieving smaller particle sizes and producing a stable nanoemulsion system^31^. Furthermore, repeated microfluidic passes likely disrupt the gel-like network structure of sodium alginate, leading to the formation of a dense matrix of alginate surrounding the oil droplets. The resulting droplet size is also influenced by the fatty acid profile of the oil phase, including chain length, degree of unsaturation, and the interfacial adsorption behavior of the emulsifier^32^. The plots of droplet intensity and volume further confirmed that multimodal patterns until the third microfluidic passes (Fig. 2), followed by a unimodal pattern, which showed the uniformity of droplets after the third microfluidic pass.

During storage, sodium alginate may undergo hydrolysis, molecular rearrangement, compaction, and partial degradation^24^. These structural changes increase steric stabilization around oil droplets, reducing the risk of coalescence. In addition, processes such as droplet dispersion, Ostwald ripening, and droplet shrinkage may further contribute to the modulation of particle size over time^33^.

### Zeta potential

The surface charge of emulsion droplets is typically assessed through zeta potential measurements. A higher absolute value of zeta potential (> 30 mV) indicates stronger electrostatic repulsion between droplets, which helps prevent flocculation, coalescence, and aggregation, thereby enhancing emulsion stability^33^.

In this study, zeta potential values were significantly (*p* < 0.05) influenced by oil type, microfluidic passes, and storage time (main effects). The zeta potential values of coconut oil and testa oil emulsions were significantly (*p* < 0.05) lower than those of the emulsions made using corn oil. The adsorbed carboxylate (COO -) groups of the sodium alginate at the oil-water interface were the main sources of the negative zeta potential of all emulsions. Coconut oil emulsions and testa oil emulsions had greater negative values of zeta potential than the corn oil emulsions. The effect is independent of the length of triglyceride chains, but it is based on the variation of droplet dimension and interfacial structure^22^. According to Hasenhuettl & Hartel^34^; Kralova & Sjöblom^1^, emulsifiers tend to adsorb preferentially over less surface-active molecules. Therefore, oil composition can indirectly affect the surface charge by influencing the competitive adsorption behaviour at the droplet interface.

The number of microfluidic passes also significantly contributed to the reduction of (more negative) zeta potential values (Table 2). As droplet size decreased through repeated processing, the number of terminal COO⁻ groups exposed to the continuous phase increased, enhancing the net negative charge of the colloidal system. In addition, mechanical disruption can alter the surface characteristics of oil droplets, increasing their capacity for stabilizing interactions with emulsifiers and alginates^32^.

Storage time had a similar effect on zeta potential. As particle shrinkage and interfacial rearrangements occurred, a higher negative surface charge was observed. However, the relatively low zeta potential values ( < ± 30 mV) indicate that electrostatic repulsion is not the dominant stabilization mechanism in these emulsions. Instead, the stability of the system is primarily associated with the structuring and steric stabilization provided by sodium alginate, which forms a network in the continuous phase and limits droplet mobility. It was further confirmed by Garti and Leser^35^ that certain hydrocolloids, such as sodium alginate, can acquire surface activity under specific conditions, further contributing to the interfacial charge and stability of emulsions^35^. A significant interaction effect between oil type x microfluidic passes x storage time (*p* < 0.05) on zeta potential was observed (Fig. 3). This may be explained by differences in the ionic composition and interfacial behavior of the oils, as previous studies have shown that ionic and surface-active compounds within oils can modify the surface charge of droplets^32^.

### Whiteness index

The color of an emulsion is its first perceived sensory attribute and is directly relevant to food applications. In this study, oil type significantly influenced the whiteness index (WI) of the emulsions. Emulsions prepared with testa oil exhibited a significantly lower particle size (*p* < 0.05) compared to those with coconut and corn oil^36^. The negative correlation of droplet size and whiteness index further confirmed that larger droplets cause stronger light scattering, they typically results in a more opaque and lower whiteness index^37^. Moreover, the reduction of droplet size decreases the turbidity of the emulsion to make a translucent system^38^. However, the optical properties of an emulsion not only depend on the mean particle size of the emulsion but also on the size distribution and refractive index of the emulsion^39^.

The lower particle size containing testa oil emulsion produced a more uniform and stable emulsion, with sodium alginate, which had significantly (*p* < 0.05) higher whiteness index than corn oil and coconut oil (Table 2). It confirmed that a mixture of factors influences the whiteness index of emulsion, not just the volume of the droplets as mentioned by previous research^40^. Similar trends have been observed in emulsions formulated with different essential oils, where variations in particle size due to oil type directly affected whiteness^9^.

Microfluidic passes also influenced the WI of the emulsions. As the number of passes increased, the size of both oil droplets and the length of the sodium alginate polymer was reduced due to higher shear forces. Typically, smaller droplet sizes lead to reduced light scattering and thus resulted a higher WI. Therefore, in this study, the WI increased slightly with microfluidization. These findings align with previous observations that nanoemulsions, although generally more transparent due to limited light scattering, can still appear optically white when the system is highly stable and well-dispersed^41,13^.

Additionally, whiteness increased over the storage period, which may be attributed to gradual reductions in particle size resulting from processes such as sedimentation, creaming, and droplet shrinkage. The interaction effects of oil type, microfluidic passes, and storage time were reflected in variations in whiteness across treatments (Supplementary Table S1).

### Creaming index

Emulsions are thermodynamically unstable but can remain kinetically stable for extended periods ranging from months to even years. The emulsifiers in the colloidal system act as a covering layer for droplets to restrict their merging together. However, droplets not adequately covered are prone to destabilization processes such as flocculation, coalescence, sedimentation, and creaming. As droplets flocculate, they may eventually combine to form larger droplets^42,39^. If these droplets have a lower density than the surrounding aqueous phase, as is the case with most edible oils, they migrate upward, resulting in creaming^22^. At day 0, emulsions that had not undergone microfluidization (passes 0) exhibited a Creaming Index (CI) of approximately 4–5%, regardless of oil type. However, even a single microfluidic pass significantly (*p* < 0.05) reduced CI to nearly 0%, indicating improved homogeneity and droplet distribution. No significant differences in CI were observed between emulsions subjected to 1 to 5 microfluidic passes, suggesting that the stabilizing effect of microfluidization plateaued after the first cycle.

After 12 days of storage, a slight increase in CI was observed in non-microfluidized emulsions, especially in those prepared with corn and testa oil, which showed a significantly (*p* < 0.05) higher CI compared to the coconut oil emulsion. The lower CI in the coconut oil system may be related to its higher content of saturated fatty acids, which could improve packing and interfacial film rigidity, enhancing emulsion stability under gravitational stress. Nevertheless, emulsions processed with at least one microfluidic pass remained fully stable, maintaining CI values close to 0%.

By day 24, all emulsions that had undergone microfluidization (≥ 1 pass) retained their stability, while non-microfluidized samples continued to exhibit minor but significant creaming. These results confirm that microfluidization is highly effective in enhancing the kinetic stability of nanoemulsions, with even a single pass being sufficient to prevent creaming over 24 days of storage.The improved stability can be attributed to the reduction in droplet size and more uniform size distribution achieved through high-pressure homogenization, as well as the synergistic effect of sodium alginate and Tween 80 in forming a strong interfacial layer around oil droplets even at low zeta potential values.

Moreover, polysaccharides contribute to the stability and rheological properties of colloidal systems by reducing flocculation, coalescence, and creaming^12^. Their type and concentration play a critical role in emulsion stability. They form a weak gel-like matrix that limits molecular mobility, but if not adsorbed onto oil droplets, flocculation may still occur (McClements, 2005). Previous research showed that adding sodium alginate after emulsion formation leads to weaker networks and increased droplet aggregation compared to co-dispersion with the emulsifier^43^.

### Rheological properties of emulsion

#### Flow properties

The efficiency of droplet disruption via microfluidization and the long-term stability of emulsions, as reflected by the creaming index, are closely linked to their rheological properties. Both the type of oil and the microfluidic passes had a significant effect (*p* < 0.05) on the emulsion viscosity and shear rate (Table 3). At low shear rate (5 s⁻¹), both shear stress and apparent viscosity were significantly higher (*p* < 0.05) in emulsions stabilized with testa and coconut oil compared to corn oil. At high shear rate (7760 s⁻¹), the highest shear stress and viscosity were again observed for emulsions with testa oil (24.909 Pa and 0.0032 Pa.s, respectively), significantly higher than corn and coconut oil systems (Table 3). This suggests a more structured internal network in emulsions with more complex lipid matrices. Microfluidization reduced both values significantly, confirming its role in breaking down droplet aggregates and improving flow. Increasing the number of microfluidic passes significantly reduced both parameters, reaching the lowest values after 4–5 passes. Overall, changes in viscosity were influenced by droplet size distribution, particle concentration, and interparticle interactions^40^.These findings align with previous studies reporting viscosity reduction following microfluidization^9^.

The consistency index (k) values followed a similar pattern (Table 3). Corn oil emulsions showed the highest consistency index (0.0059 Pa.s), while testa oil resulted in the lowest values (0.0042 Pa.s). Increased microfluidization reduced the consistency index significantly from 0.0062 Pa.s (0 passes) to 0.0043 Pa.s (4 passes). A significant decrease was also observed over storage, with k-value dropping from 0.0053 Pa.s (day 0) to 0.0049 Pa.s (day 24), suggesting structure weakening or partial destabilization. In contrast, the flow behaviour index (n) increased slightly with both microfluidization and storage, indicating a transition toward more Newtonian-like behaviour (Table 3). Emulsions with testa oil showed the highest n values (0.975), suggesting lower shear-thinning characteristics. The highest n values were also recorded after 3–5 microfluidic passes and at 24 days of storage (0.959), supporting the hypothesis of structural simplification over time. The yield stress (τ₀) values, described by the Herschel–Bulkley model, were significantly higher for emulsions with testa oil (0.087) compared to coconut and corn oils (both ~ 0.050 Pa) (Table 3). Interestingly, yield stress increased after 1 microfluidization cycle (0.108 Pa), likely due to droplet rearrangement and transient structure formation, but then decreased with further processing. Storage time increased τ₀ significantly from 0.032 Pa (day 0) to 0.094 Pa (day 12), followed by a slight decline to 0.067 Pa at day 24, which may indicate structural ageing followed by partial collapse.

The observed increase in shear stress alongside a decrease in apparent viscosity with increasing shear rate confirms the pseudoplastic (shear-thinning) behaviour of the emulsions. This non-Newtonian flow characteristic is typical for structured fluid systems, where internal interactions such as droplet–droplet associations or alginate networks are progressively disrupted under shear. As a result, less force is needed per unit of shear rate to maintain flow, hence the reduction in viscosity.

Such behaviour is advantageous in practical applications, as it allows the emulsions to maintain structural integrity at rest, while becoming easily spreadable or pumpable under stress. This rheological profile is desirable for food and cosmetic emulsions, where both stability and sensory or processing functionality are important.

#### Thixotropic properties

**Fig. 4:**
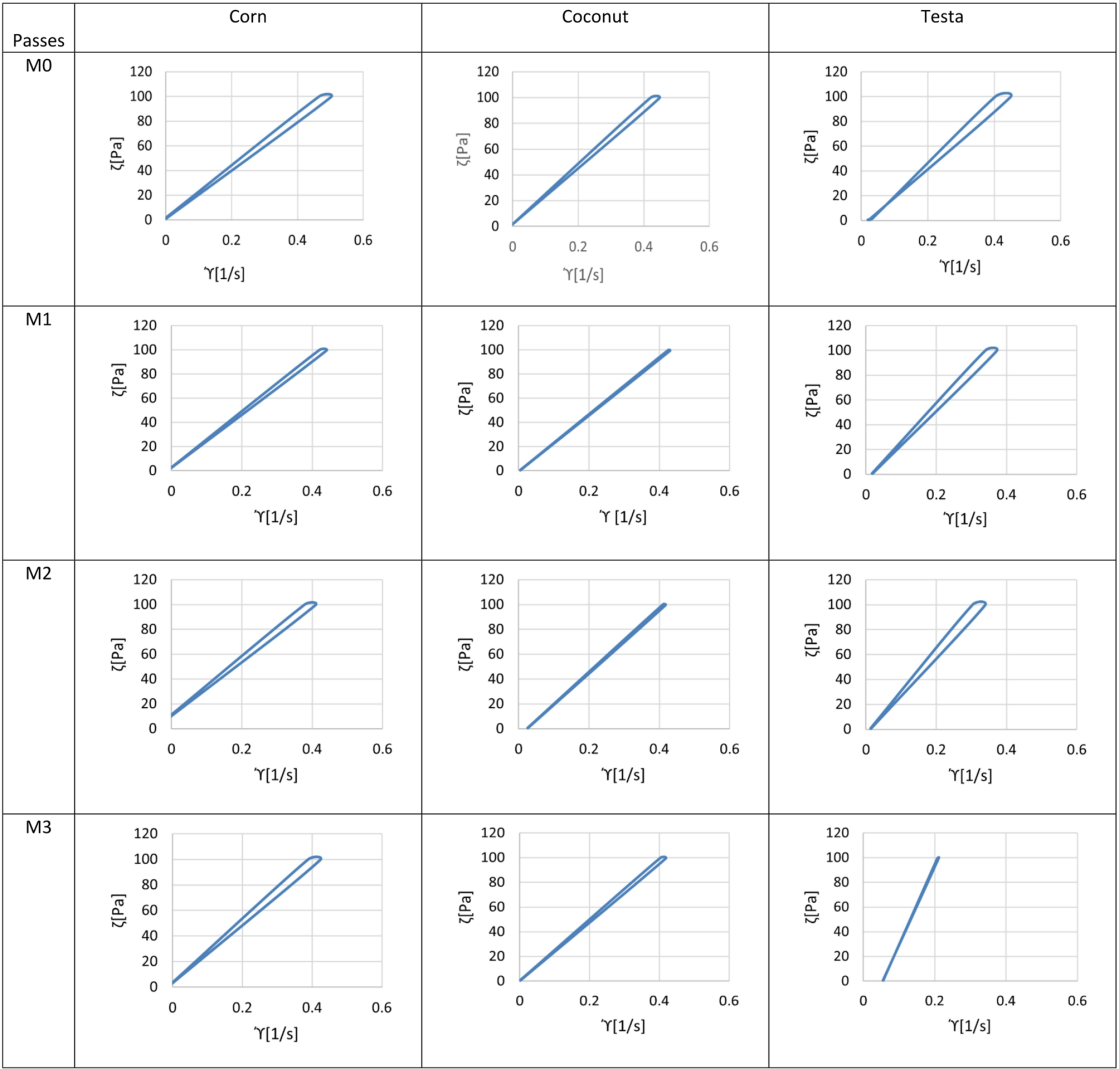

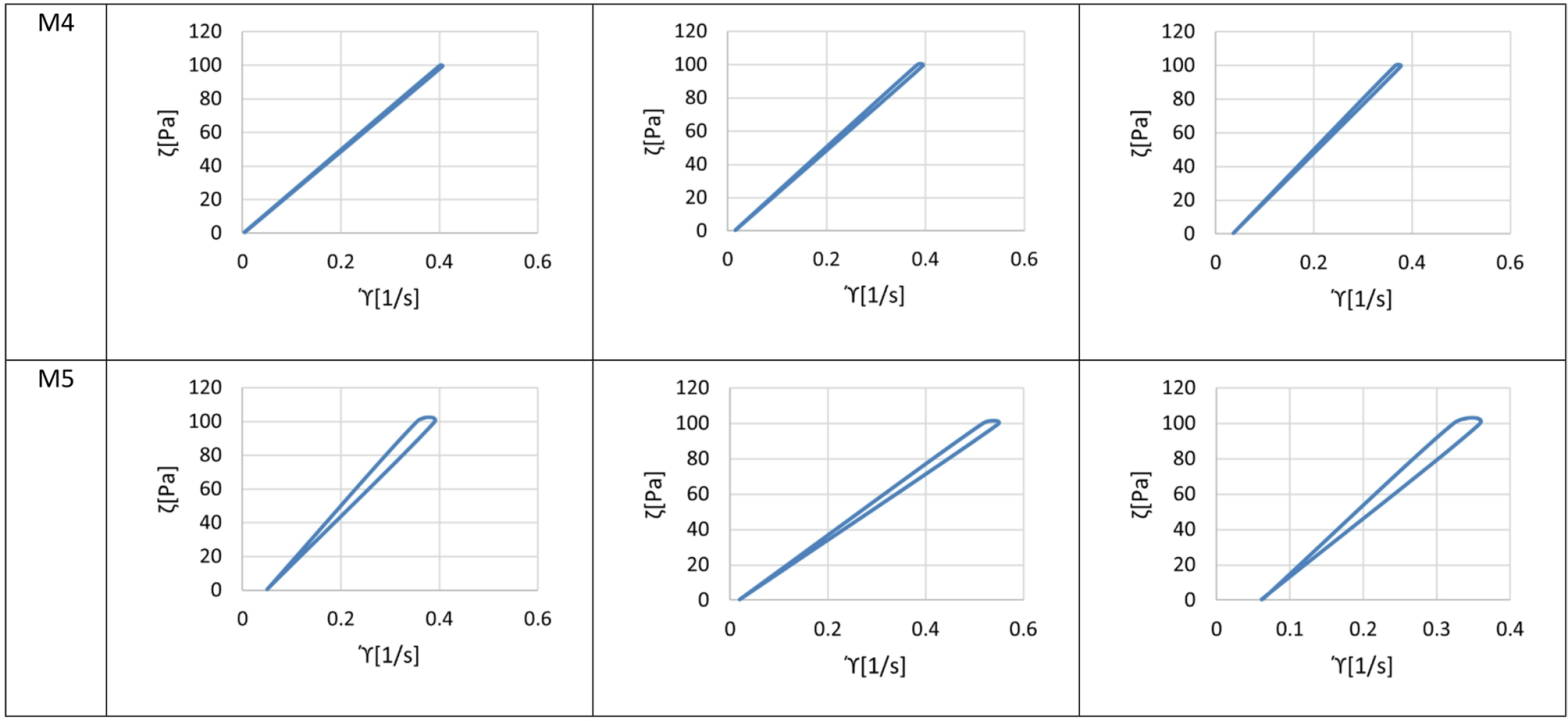
Changes in the thixotropy area (interaction effect) of emulsions prepared with three oil types and microfluidic passes at the initial storage time (0 days).

The thixotropy area reflects the ability of an emulsion to recover its internal structure after shear. As shown in Table 3, both the main effect of oil type and microfluidic passes significantly (*p* < 0.05) affected this parameter, while storage time had no significant influence (*p* > 0.05). Therefore, the thixotropic areas of the initial emulsions are shown in Fig. 4. Among the oil types, emulsions prepared with testa oil exhibited the largest thixotropy area (7.10 Pa/s), indicating the lowest ability to rebuild internal structure after shear stress. In contrast, corn oil emulsions had the smallest thixotropy area (4.57 Pa/s), suggesting better structural recovery. This may be linked to the fatty acid composition and molecular interactions within the emulsion matrix.

Microfluidization passes also affected thixotropy in a non-linear manner. The lowest area was observed after 1 pass (5.36 Pa/s), while the highest occurred after 2 passes (6.71 Pa/s). No consistent trend was observed across the remaining passes. These fluctuations may result from dynamic changes in the alginate droplet network under increasing shear and pressure.

Storage time (0, 12, 24 days) did not significantly (*p* > 0.05) influence the thixotropic area, suggesting that the structural recovery behaviour remained stable over the tested period.

#### Viscoelastic properties

The viscoelastic properties of the emulsions did not differ significantly (*p* > 0.05) across the main effects (oil type, microfluidic passes, and storage time) (Table 3) or their interactions (Supplementary Table S1). The average values of storage modulus (G′), loss modulus (G″), and loss tangent (tan δ) were 18.73, 2.83, and 0.69, respectively.

Since the ratio G″/G′ remained below 1 for all samples, the emulsions exhibited predominantly elastic behavior, characteristic of weak gel-like structures. This suggests that the internal network of the emulsions was dominated by elastic (solid-like) components, yet not strong enough to be classified as true gels.

### Principal component analysis

**Fig. 5:**
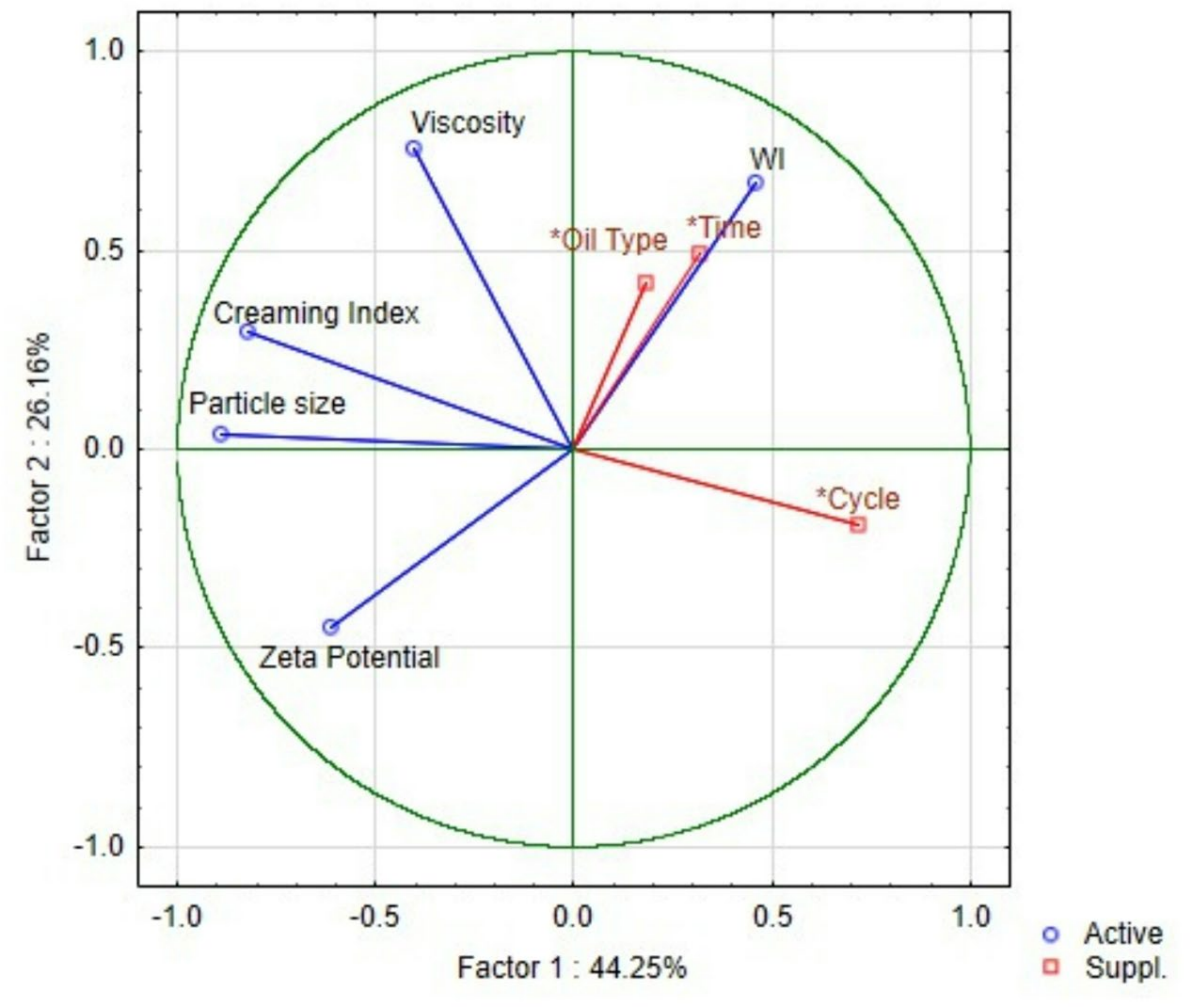
Principal component analysis of each parameter with the three main effects of oil type, microfluidic passes and storage tim.

To explore the relationships between processing factors and emulsion characteristics, principal component analysis (PCA) was performed (Fig. 5). The first two principal components (PC1 and PC2) explained 44.25% and 26.16% of the total variance, respectively.

Among the variables, Whiteness Index (WI) and viscosity were strongly and positively associated with Factor 1, whereas zeta potential, particle size, and creaming index were negatively correlated with this axis. These groupings suggest that smaller droplets with higher surface charge (lower zeta potential) were associated with lower CI and occurred under different conditions than those yielding high WI and viscosity.

Regarding the supplementary variables (in red), microfluidic passes was the most strongly associated with zeta potential, particle size, and creaming index, implying that increasing the number of microfluidic passes reduced droplet size and improved colloidal stability. On the other hand, oil type and storage time aligned more closely with WI and viscosity, suggesting these attributes were more influenced by formulation composition and ageing than by processing intensity.

Overall, PCA effectively separated the effects of formulation (oil type), processing (microfluidic passes), and storage (storage time) on emulsion characteristics, highlighting microfluidic passesas a major factor influencing physicochemical stability.

## Conclusions

Microfluidization proved to be an effective strategy for controlling emulsion structure and stability, with the most improvements obtained after three passes. Increasing the number of passes significantly reduced droplet size and zeta potential. However, the relatively low zeta potential indicated that steric stabilisation of the sodium alginate network in the continuous phase improved the stability of the emulsion. These structural modifications resulted in a substantial reduction of the creaming index, particularly after the initial microfluidic pass.

The Whiteness Index was primarily governed by oil type, microfluidic passes and storage time, and higher values observed for testa oil emulsions than other oil types.Although microfluidization induced partial alginate fragmentation and a concomitant decrease in viscosity, all emulsions maintained viscoelastic behaviour (G′ > G″), characteristic of weak gel-like systems, throughout storage. Importantly, these physicochemical changes supported structural rearrangements during ageing without compromising overall stability. Oil type significantly influenced rheological and optical properties. Testa oil produced emulsions with the highest viscosity and thixotropy but exhibited weaker structural recovery. Corn oil systems demonstrated more favourable flow behaviour and lower thixotropic area, whereas coconut oil displayed intermediate characteristics. Principal Component Analysis confirmed that formulation variables predominantly determined viscosity and color parameters, while the number of microfluidic passes was the key driver of stability-related attributes, including particle size, zeta potential, and creaming index.

## Supplementary Information

Below is the link to the electronic supplementary material.

Supplementary Material 1

## Data Availability

Data is available on request.
