# Supplementary material for: Comparative evaluation of corn, coconut, and coconut testa oils in sodium alginate-stabilized nanoemulsions processed by microfluidization
Source: Sci Rep. 2026 Apr 26;16:19245. doi: 10.1038/s41598-026-49545-5 (PMC13284227; doi:10.1038/s41598-026-49545-5)
Supplement: Supplementary file 1 — Supplementary Material 1 [file 41598_2026_49545_MOESM1_ESM.docx]

| No | Oil type | Microfluidic passes | Storage time (days) | pH | EC [µS/cm] | L* | a* | b* | Z average Particle size [nm] | Polydispersity Index  (PDI) |
| --- | --- | --- | --- | --- | --- | --- | --- | --- | --- | --- |
| 1 | Corn | M0 | T0 | 5.55±0.05 | 1102.00±5.77 | 45.66±2.47 | 0.44±0.07 | 8.39±1.02 | 508.80±84.18 | 0.91±0.09 |
| 2 | Corn | M0 | T12 | 6.02±0.09 | 1122.50±0.58 | 37.92±0.64 | 0.50±0.19 | 9.01±0.34 | 414.00±29.44 | 0.46±0.05 |
| 3 | Corn | M0 | T24 | 5.94±0.13 | 1148.50±2.89 | 46.29±7.56 | 0.90±0.08 | 0.84±0.70 | 206.65±5.48 | 0.56±0.04 |
| 4 | Corn | M1 | T0 | 5.59±0.05 | 1110.50±2.89 | 45.61±3.62 | 1.59±0.28 | 12.18±0.55 | 291.85±53.06 | 0.52±0.12 |
| 5 | Corn | M1 | T12 | 5.99±0.03 | 1117.00±9.24 | 42.90±2.83 | 1.23±0.28 | 13.29±0.61 | 211.25±0.06 | 0.35±0.05 |
| 6 | Corn | M1 | T24 | 5.74±0.06 | 1131.50±13.28 | 48.86±3.18 | 1.52±0.33 | 10.42±2.03 | 216.05±9.41 | 0.40±0.01 |
| 7 | Corn | M2 | T0 | 5.72±0.03 | 1112.00±3.46 | 38.87±1.91 | 2.10±0.14 | 8.77±0.40 | 187.90±8.43 | 0.44±0.02 |
| 8 | Corn | M2 | T12 | 5.89±0.08 | 1116.50±0.58 | 38.76±0.34 | 2.59±0.11 | 7.00±0.19 | 178.90±3.81 | 0.46±0.01 |
| 9 | Corn | M2 | T24 | 5.78±0.08 | 1137.50±4.04 | 52.27±1.95 | 2.97±0.14 | 2.53±0.78 | 170.15±4.79 | 0.43±0.00 |
| 10 | Corn | M3 | T0 | 5.69±0.05 | 1118.50±4.04 | 37.22±1.62 | 3.08±0.08 | 3.73±1.53 | 130.85±7.10 | 0.41±0.02 |
| 11 | Corn | M3 | T12 | 5.98±0.05 | 1121.50±2.89 | 45.28±1.18 | 3.96±0.08 | 1.88±1.82 | 124.70±0.46 | 0.40±0.01 |
| 12 | Corn | M3 | T24 | 5.92±0.04 | 1115.50±1.73 | 49.76±5.76 | 3.52±0.15 | 1.26±1.47 | 167.95±51.67 | 0.39±0.00 |
| 13 | Corn | M4 | T0 | 5.54±0.02 | 1114.50±1.73 | 37.56±0.36 | 4.83±0.03 | 0.70±1.76 | 100.21±1.96 | 0.36±0.01 |
| 14 | Corn | M4 | T12 | 6.00±0.01 | 1113.50±4.04 | 43.55±1.91 | 4.65±0.20 | 1.79±0.90 | 99.75±0.00 | 0.35±0.01 |
| 15 | Corn | M4 | T24 | 5.83±0.01 | 1110.50±1.73 | 55.67±4.74 | 4.09±0.18 | 4.91±0.85 | 99.59±0.82 | 0.32±0.03 |
| 16 | Corn | M5 | T0 | 5.61±0.05 | 1119.00±2.31 | 37.08±0.76 | 5.52±0.22 | 1.05±0.75 | 89.08±3.57 | 0.27±0.00 |
| 17 | Corn | M5 | T12 | 6.14±0.08 | 1121.00±5.77 | 44.05±2.48 | 5.28±0.03 | 3.82±0.70 | 89.72±1.59 | 0.27±0.00 |
| 18 | Corn | M5 | T24 | 5.89±0.05 | 1122.50±9.81 | 49.44±3.92 | 5.57±0.04 | 4.38±0.35 | 88.29±1.25 | 0.30±0.05 |
| 19 | Coconut | M0 | T0 | 5.59±0.09 | 1112.00±2.31 | 40.38±1.39 | 0.06±0.11 | 10.99±0.68 | 382.95±58.83 | 0.61±0.10 |
| 20 | Coconut | M0 | T12 | 5.98±0.02 | 1127.50±4.04 | 39.08±2.96 | 0.00±0.14 | 9.36±1.29 | 267.70±19.98 | 0.46±0.13 |
| 21 | Coconut | M0 | T24 | 5.90±0.07 | 1140.50±15.59 | 48.63±1.46 | 0.29±0.05 | 2.73±2.27 | 229.20±31.87 | 0.57±0.07 |
| 22 | Coconut | M1 | T0 | 6.06±0.23 | 1120.50±2.89 | 36.53±0.94 | 1.60±0.06 | 10.41±0.45 | 208.85±40.13 | 0.49±0.07 |
| 23 | Coconut | M1 | T12 | 6.18±0.39 | 1151.00±3.46 | 40.63±3.09 | 1.66±0.12 | 7.03±1.52 | 165.95±3.64 | 0.45±0.07 |
| 24 | Coconut | M1 | T24 | 6.20±0.29 | 1160.00±4.62 | 53.31±1.68 | 1.46±0.45 | 1.70±2.61 | 174.05±13.22 | 0.41±0.02 |
| 25 | Coconut | M2 | T0 | 5.64±0.04 | 1116.00±3.46 | 34.85±0.29 | 2.69±0.05 | 2.02±0.09 | 160.85±27.54 | 0.43±0.01 |
| 26 | Coconut | M2 | T12 | 5.92±0.17 | 1141.50±4.04 | 41.33±2.65 | 2.56±0.35 | 0.61±1.59 | 132.00±7.62 | 0.41±0.01 |
| 27 | Coconut | M2 | T24 | 5.77±0.01 | 1155.50±0.58 | 50.28±0.31 | 2.34±0.24 | 1.55±2.24 | 118.50±0.92 | 0.39±0.02 |
| 28 | Coconut | M3 | T0 | 5.69±0.01 | 1120.00±2.31 | 34.93±0.36 | 3.48±0.09 | 0.15±0.54 | 118.35±7.33 | 0.42±0.03 |
| 29 | Coconut | M3 | T12 | 5.88±0.15 | 1136.50±4.04 | 43.73±2.83 | 3.05±0.49 | 2.72±0.75 | 101.11±1.84 | 0.36±0.02 |
| 30 | Coconut | M3 | T24 | 5.83±0.05 | 1112.50±12.12 | 57.24±0.33 | 3.63±0.32 | 5.27±0.85 | 100.89±4.86 | 0.37±0.04 |
| 31 | Coconut | M4 | T0 | 5.68±0.09 | 1119.50±1.73 | 35.10±0.79 | 4.42±0.36 | 0.19±0.65 | 100.78±1.88 | 0.36±0.06 |
| 32 | Coconut | M4 | T12 | 5.81±0.13 | 1138.00±3.46 | 43.90±3.34 | 4.06±0.56 | 3.50±1.59 | 123.85±22.46 | 0.46±0.14 |
| 33 | Coconut | M4 | T24 | 5.81±0.18 | 1120.50±19.05 | 58.23±2.10 | 4.57±0.44 | 4.86±0.97 | 94.32±12.33 | 0.36±0.03 |
| 34 | Coconut | M5 | T0 | 5.63±0.12 | 1123.50±1.73 | 34.39±0.52 | 5.71±0.10 | 2.09±1.27 | 91.00±2.55 | 0.34±0.07 |
| 35 | Coconut | M5 | T12 | 5.79±0.06 | 1145.00±1.15 | 42.36±1.44 | 5.04±0.60 | 2.81±1.11 | 93.97±12.97 | 0.34±0.01 |
| 36 | Coconut | M5 | T24 | 5.80±0.06 | 1113.50±4.04 | 58.42±4.72 | 5.64±0.47 | 4.99±1.20 | 91.95±10.34 | 0.33±0.02 |
| 37 | Testa | M0 | T0 | 5.56±0.03 | 1137.00±3.46 | 46.03±1.85 | 0.39±0.03 | 10.66±0.37 | 278.80±10.74 | 0.68±0.02 |
| 38 | Testa | M0 | T12 | 5.33±0.01 | 1112.00±1.15 | 50.25±4.32 | 0.26±0.40 | 12.94±0.94 | 245.45±10.33 | 0.54±0.12 |
| 39 | Testa | M0 | T24 | 5.50±0.16 | 1123.50±2.89 | 46.42±2.93 | 0.95±0.32 | 8.61±3.14 | 186.80±1.39 | 0.36±0.06 |
| 40 | Testa | M1 | T0 | 5.34±0.21 | 1135.00±8.08 | 53.78±2.49 | 1.70±0.03 | 2.62±5.37 | 190.15±0.06 | 0.44±0.01 |
| 41 | Testa | M1 | T12 | 5.21±0.00 | 1122.00±3.46 | 56.38±1.59 | 1.58±0.17 | 6.08±1.03 | 181.65±2.83 | 0.43±0.01 |
| 42 | Testa | M1 | T24 | 5.58±0.10 | 1122.00±0.00 | 54.65±4.66 | 1.08±0.16 | 2.88±1.43 | 164.20±4.62 | 0.42±0.01 |
| 43 | Testa | M2 | T0 | 5.48±0.03 | 1135.50±5.20 | 54.89±1.42 | 2.64±0.36 | 2.82±2.60 | 131.90±0.23 | 0.42±0.01 |
| 44 | Testa | M2 | T12 | 5.31±0.17 | 1131.50±1.73 | 59.66±3.00 | 2.53±0.08 | 1.05±0.46 | 128.15±6.41 | 0.43±0.02 |
| 45 | Testa | M2 | T24 | 5.76±0.12 | 1113.50±0.58 | 49.97±4.32 | 2.60±0.13 | 2.91±0.44 | 122.25±3.75 | 0.38±0.00 |
| 46 | Testa | M3 | T0 | 5.53±0.02 | 1139.50±0.58 | 58.28±2.24 | 3.41±0.11 | 0.31±2.22 | 111.75±6.41 | 0.39±0.04 |
| 47 | Testa | M3 | T12 | 5.37±0.05 | 1093.00±6.93 | 56.37±4.88 | 3.23±0.13 | 0.69±1.03 | 110.25±6.75 | 0.39±0.03 |
| 48 | Testa | M3 | T24 | 5.37±0.07 | 1112.50±12.12 | 61.13±5.05 | 3.48±0.17 | 1.74±0.64 | 101.95±0.29 | 0.36±0.01 |
| 49 | Testa | M4 | T0 | 5.58±0.04 | 1114.00±33.49 | 59.67±1.24 | 4.10±0.12 | 2.84±0.86 | 97.59±0.77 | 0.31±0.02 |
| 50 | Testa | M4 | T12 | 5.42±0.17 | 1098.00±1.15 | 59.02±0.84 | 4.96±0.09 | 0.83±0.60 | 94.28±0.13 | 0.30±0.04 |
| 51 | Testa | M4 | T24 | 5.34±0.04 | 1132.50±1.73 | 64.90±4.07 | 4.28±0.17 | 1.92±0.75 | 91.30±0.53 | 0.30±0.01 |
| 52 | Testa | M5 | T0 | 5.59±0.04 | 1121.00±17.32 | 56.38±2.94 | 5.45±0.10 | 1.42±1.71 | 98.35±1.31 | 0.33±0.04 |
| 53 | Testa | M5 | T12 | 5.47±0.12 | 1110.00±0.00 | 58.35±2.86 | 5.79±0.15 | 3.19±0.53 | 97.89±6.14 | 0.38±0.20 |
| 54 | Testa | M5 | T24 | 5.17±0.04 | 1129.50±1.73 | 65.41±3.61 | 5.20±0.10 | 2.45±0.12 | 89.37±0.62 | 0.27±0.00 |

Table S1: Mean value with standard deviation of three way interaction of each factor of oil type, microfluidic passes and storage time for all parameters.

| No | Oil type | Microfluidic passes | Storage Time | Zeta Potential [mV] | Creaming Index [%] | Shear stress 5s-1 [Pa] | Shear stress 7760s-1 [Pa] | Apparent Viscosity 5s-1 [Pa.s] | Apparent Viscosity 7760s-1  [Pa.s] |  |
| --- | --- | --- | --- | --- | --- | --- | --- | --- | --- | --- |
| 1 | Corn | M0 | T0 | 2.27±0.04 | 4.00±0.00 | 0.08±0.03 | 25.49±1.65 | 0.015±0.006 | 0.00329±0.00021 |  |
| 2 | Corn | M0 | T12 | 6.35±0.42 | 5.00±0.00 | 0.06±0.02 | 26.52±0.83 | 0.011±0.003 | 0.00342±0.00011 |  |
| 3 | Corn | M0 | T24 | 9.12±0.57 | 5.00±0.00 | 0.15±0.08 | 24.38±2.91 | 0.029±0.016 | 0.00315±0.00038 |  |
| 4 | Corn | M1 | T0 | 4.87±0.47 | 0.00±0.00 | 1.04±1.01 | 21.97±0.74 | 0.008±1.001 | 0.00284±0.00010 |  |
| 5 | Corn | M1 | T12 | 7.91±1.86 | 0.00±0.00 | 1.04±1.00 | 24.66±2.03 | 0.007±1.000 | 0.00318±0.00026 |  |
| 6 | Corn | M1 | T24 | 10.01±0.06 | 0.00±0.00 | 1.07±1.04 | 22.15±2.54 | 0.014±1.009 | 0.00286±0.00033 |  |
| 7 | Corn | M2 | T0 | 5.59±0.49 | 0.00±0.00 | 2.04±2.01 | 22.22±0.97 | 0.008±2.001 | 0.00287±0.00013 |  |
| 8 | Corn | M2 | T12 | 6.78±0.00 | 0.00±0.00 | 2.06±2.03 | 20.79±0.53 | 0.013±2.006 | 0.00268±0.00007 |  |
| 9 | Corn | M2 | T24 | 9.92±0.57 | 0.00±0.00 | 2.06±2.01 | 23.82±1.64 | 0.012±2.002 | 0.00308±0.00021 |  |
| 10 | Corn | M3 | T0 | 6.70±1.01 | 0.00±0.00 | 3.03±3.00 | 20.80±1.04 | 0.007±3.000 | 0.00268±0.00013 |  |
| 11 | Corn | M3 | T12 | 8.30±0.38 | 0.00±0.00 | 3.04±3.02 | 20.39±0.98 | 0.008±3.003 | 0.00263±0.00013 |  |
| 12 | Corn | M3 | T24 | 10.81±0.53 | 0.00±0.00 | 3.07±3.04 | 24.42±2.36 | 0.013±3.008 | 0.00315±0.00030 |  |
| 13 | Corn | M4 | T0 | 8.12±1.26 | 0.00±0.00 | 4.04±4.00 | 22.20±0.29 | 0.007±4.000 | 0.00287±0.00004 |  |
| 14 | Corn | M4 | T12 | 10.68±0.53 | 0.00±0.00 | 4.03±4.01 | 21.96±0.43 | 0.006±4.002 | 0.00283±0.00006 |  |
| 15 | Corn | M4 | T24 | 11.99±0.11 | 0.00±0.00 | 4.04±4.01 | 20.70±1.45 | 0.007±4.002 | 0.00267±0.00019 |  |
| 16 | Corn | M5 | T0 | 7.48±1.43 | 0.00±0.00 | 5.07±5.04 | 20.77±1.23 | 0.014±5.008 | 0.00268±0.00016 |  |
| 17 | Corn | M5 | T12 | 14.15±2.89 | 0.00±0.00 | 5.04±5.02 | 23.40±0.85 | 0.008±5.004 | 0.00302±0.00011 |  |
| 18 | Corn | M5 | T24 | 14.63±2.18 | 0.00±0.00 | 5.03±5.00 | 25.26±0.01 | 0.007±5.001 | 0.00326±0.00000 |  |
| 19 | Coconut | M0 | T0 | 5.29±0.90 | 4.00±0.00 | 0.08±0.05 | 23.10±5.05 | 0.017±0.009 | 0.00298±0.00065 |  |
| 20 | Coconut | M0 | T12 | 6.77±0.96 | 4.00±0.00 | 0.12±0.02 | 26.54±0.12 | 0.023±0.004 | 0.00343±0.00001 |  |
| 21 | Coconut | M0 | T24 | 10.92±0.86 | 5.00±0.00 | 0.15±0.06 | 26.14±0.58 | 0.030±0.003 | 0.00337±0.00008 |  |
| 22 | Coconut | M1 | T0 | 9.93±2.75 | 0.00±0.00 | 1.13±1.04 | 17.50±10.24 | 0.026±1.009 | 0.00226±0.00032 |  |
| 23 | Coconut | M1 | T12 | 11.27±1.36 | 0.00±0.00 | 1.21±1.05 | 22.69±1.65 | 0.042±1.000 | 0.00293±0.00021 |  |
| 24 | Coconut | M1 | T24 | 13.05±1.39 | 0.00±0.00 | 1.08±1.00 | 23.57±1.40 | 0.016±1.001 | 0.00304±0.00018 |  |
| 25 | Coconut | M2 | T0 | 6.73±1.76 | 0.00±0.00 | 2.07±2.01 | 22.51±1.43 | 0.014±2.002 | 0.00291±0.00019 |  |
| 26 | Coconut | M2 | T12 | 10.99±0.44 | 0.00±0.00 | 2.23±2.05 | 20.78±0.84 | 0.046±2.000 | 0.00268±0.00011 |  |
| 27 | Coconut | M2 | T24 | 13.20±0.16 | 0.00±0.00 | 2.07±2.03 | 22.91±0.29 | 0.013±2.007 | 0.00296±0.00004 |  |
| 28 | Coconut | M3 | T0 | 7.16±0.84 | 0.00±0.00 | 3.06±3.04 | 22.02±1.59 | 0.013±3.008 | 0.00284±0.00021 |  |
| 29 | Coconut | M3 | T12 | 11.55±0.64 | 0.00±0.00 | 3.18±3.05 | 20.98±0.70 | 0.037±3.001 | 0.00271±0.00009 |  |
| 30 | Coconut | M3 | T24 | 11.90±0.20 | 0.00±0.00 | 3.04±3.03 | 24.48±0.76 | 0.009±3.005 | 0.00316±0.00010 |  |
| 31 | Coconut | M4 | T0 | 5.58±1.05 | 0.00±0.00 | 4.09±4.07 | 15.90±7.89 | 0.018±4.003 | 0.00205±0.00002 |  |
| 32 | Coconut | M4 | T12 | 12.23±0.67 | 0.00±0.00 | 4.15±4.06 | 22.58±1.17 | 0.030±4.001 | 0.00292±0.00015 |  |
| 33 | Coconut | M4 | T24 | 11.10±2.32 | 0.00±0.00 | 4.05±4.03 | 19.68±8.93 | 0.011±4.006 | 0.00254±0.00015 |  |
| 34 | Coconut | M5 | T0 | 4.42±0.50 | 0.00±0.00 | 5.03±5.01 | 21.29±1.28 | 0.006±5.002 | 0.00275±0.00017 |  |
| 35 | Coconut | M5 | T12 | 10.68±3.19 | 0.00±0.00 | 5.26±5.07 | 21.53±0.90 | 0.051±5.004 | 0.00278±0.00012 |  |
| 36 | Coconut | M5 | T24 | 12.51±0.78 | 0.00±0.00 | 5.05±5.04 | 24.70±0.43 | 0.011±5.007 | 0.00319±0.00006 |  |
| 37 | Testa | M0 | T0 | 7.67±0.19 | 4.00±0.00 | 0.09±0.00 | 29.90±1.41 | 0.019±0.001 | 0.00386±0.00018 |  |
| 38 | Testa | M0 | T12 | 7.53±0.19 | 5.00±0.00 | 0.08±0.04 | 29.71±0.24 | 0.015±0.009 | 0.00384±0.00003 |  |
| 39 | Testa | M0 | T24 | 11.02±0.97 | 5.00±0.00 | 0.16±0.02 | 28.99±1.72 | 0.032±0.003 | 0.00374±0.00022 |  |
| 40 | Testa | M1 | T0 | 8.13±0.72 | 0.00±0.00 | 1.15±1.05 | 27.44±3.00 | 0.031±1.001 | 0.00354±0.00039 |  |
| 41 | Testa | M1 | T12 | 8.92±0.84 | 0.00±0.00 | 1.08±1.06 | 24.37±0.88 | 0.016±1.002 | 0.00315±0.00011 |  |
| 42 | Testa | M1 | T24 | 11.37±0.02 | 0.00±0.00 | 1.91±1.07 | 28.89±2.01 | 0.182±1.005 | 0.00373±0.00026 |  |
| 43 | Testa | M2 | T0 | 7.40±0.21 | 0.00±0.00 | 2.11±2.03 | 25.65±1.97 | 0.022±2.007 | 0.00331±0.00025 |  |
| 44 | Testa | M2 | T12 | 7.55±0.52 | 0.00±0.00 | 2.04±2.00 | 27.97±0.67 | 0.009±2.000 | 0.00361±0.00009 |  |
| 45 | Testa | M2 | T24 | 10.79±0.90 | 0.00±0.00 | 2.12±2.02 | 17.63±4.94 | 0.024±2.003 | 0.00228±0.00064 |  |
| 46 | Testa | M3 | T0 | 9.47±0.06 | 0.00±0.00 | 3.13±3.00 | 24.31±2.28 | 0.027±3.000 | 0.00314±0.00029 |  |
| 47 | Testa | M3 | T12 | 7.39±1.12 | 0.00±0.00 | 3.06±3.02 | 26.75±0.00 | 0.013±3.004 | 0.00345±0.00000 |  |
| 48 | Testa | M3 | T24 | 14.85±6.64 | 0.00±0.00 | 3.15±3.03 | 21.61±1.09 | 0.030±3.006 | 0.00279±0.00014 |  |
| 49 | Testa | M4 | T0 | 8.35±0.10 | 0.00±0.00 | 4.12±4.01 | 24.40±2.71 | 0.025±4.002 | 0.00315±0.00035 |  |
| 50 | Testa | M4 | T12 | 8.17±0.39 | 0.00±0.00 | 4.12±4.00 | 25.74±0.61 | 0.024±4.001 | 0.00332±0.00008 |  |
| 51 | Testa | M4 | T24 | 9.85±0.75 | 0.00±0.00 | 4.02±4.00 | 23.79±0.90 | 0.005±4.001 | 0.00307±0.00012 |  |
| 52 | Testa | M5 | T0 | 9.07±2.48 | 0.00±0.00 | 5.12±5.06 | 22.75±1.07 | 0.024±5.002 | 0.00294±0.00014 |  |
| 53 | Testa | M5 | T12 | 8.66±1.32 | 0.00±0.00 | 5.19±5.01 | 18.68±1.22 | 0.039±5.002 | 0.00241±0.00016 |  |
| 54 | Testa | M5 | T24 | 7.11±0.29 | 0.00±0.00 | 5.05±5.01 | 19.80±7.22 | 0.011±5.002 | 0.00256±0.00093 |  |

| No | Oil type | Microfluidic passes | Storage Time | Thixotropic area [Pa/s] | WI | Loss tangent (tan δ) |
| --- | --- | --- | --- | --- | --- | --- |
| 1 | Corn | M0 | T0 | 4.14±0.63 | 45.01±2.56 | 0.730±0.145 |
| 2 | Corn | M0 | T12 | 3.06±1.79 | 37.27±0.67 | 0.599±0.148 |
| 3 | Corn | M0 | T24 | .5.45±1.85 | 46.28±7.57 | 0.690±0.159 |
| 4 | Corn | M1 | T0 | 4.36±0.83 | 44.24±3.41 | 0.630±0.129 |
| 5 | Corn | M1 | T12 | 3.10±0.46 | 41.37±2.62 | 0.733±0.155 |
| 6 | Corn | M1 | T24 | 4.77±1.46 | 47.75±2.95 | 0.772±0.092 |
| 7 | Corn | M2 | T0 | 3.56±0.04 | 38.23±1.84 | 0.690±0.105 |
| 8 | Corn | M2 | T12 | 6.10±3.01 | 38.34±0.32 | 0.649±0.180 |
| 9 | Corn | M2 | T24 | 5.37±0.95 | 52.16±1.96 | 0.790±0.135 |
| 10 | Corn | M3 | T0 | 3.39±0.15 | 37.06±1.68 | 0.649±0.193 |
| 11 | Corn | M3 | T12 | 4.40±0.57 | 45.19±1.17 | 0.690±0.126 |
| 12 | Corn | M3 | T24 | 5.37±0.64 | 49.70±5.75 | 0.770±0.164 |
| 13 | Corn | M4 | T0 | 4.49±1.29 | 37.51±0.38 | 0.690±0.145 |
| 14 | Corn | M4 | T12 | 3.87±0.62 | 43.49±1.90 | 0.588±0.113 |
| 15 | Corn | M4 | T24 | 5.25±1.52 | 55.38±4.74 | 0.658±0.017 |
| 16 | Corn | M5 | T0 | 5.09±1.87 | 37.05±0.76 | 0.715±0.096 |
| 17 | Corn | M5 | T12 | 5.20±1.61 | 43.90±2.43 | 0.771±0.131 |
| 18 | Corn | M5 | T24 | 5.27±0.78 | 49.25±3.91 | 0.711±0.127 |
| 19 | Coconut | M0 | T0 | 5.32±0.57 | 39.36±1.24 | 0.698±0.274 |
| 20 | Coconut | M0 | T12 | 10.95±1.45 | 38.35±2.97 | 0.796±0.144 |
| 21 | Coconut | M0 | T24 | 6.73±1.61 | 48.50±1.43 | 0.755±0.094 |
| 22 | Coconut | M1 | T0 | 5.19±1.82 | 35.67±0.98 | 0.649±0.048 |
| 23 | Coconut | M1 | T12 | 4.05±2.11 | 40.16±2.89 | 0.650±0.279 |
| 24 | Coconut | M1 | T24 | 6.18±1.27 | 53.20±1.59 | 0.677±0.108 |
| 25 | Coconut | M2 | T0 | 8.05±5.42 | 34.79±0.29 | 0.613±0.161 |
| 26 | Coconut | M2 | T12 | 8.78±3.65 | 41.29±2.64 | 0.687±0.151 |
| 27 | Coconut | M2 | T24 | 5.58±0.29 | 50.20±0.33 | 0.657±0.109 |
| 28 | Coconut | M3 | T0 | 5.69±3.07 | 34.91±0.36 | 0.706±0.151 |
| 29 | Coconut | M3 | T12 | 5.55±2.92 | 43.65±2.81 | 0.649±0.056 |
| 30 | Coconut | M3 | T24 | 5.82±0.51 | 56.91±0.37 | 0.682±0.183 |
| 31 | Coconut | M4 | T0 | 4.10±0.42 | 35.08±0.78 | 0.759±0.112 |
| 32 | Coconut | M4 | T12 | 3.92±0.31 | 43.76±3.25 | 0.619±0.127 |
| 33 | Coconut | M4 | T24 | 5.87±0.85 | 57.94±2.08 | 0.739±0.114 |
| 34 | Coconut | M5 | T0 | 4.76±1.36 | 34.33±0.54 | 0.712±0.179 |
| 35 | Coconut | M5 | T12 | 9.91±0.91 | 42.27±1.40 | 0.729±0.081 |
| 36 | Coconut | M5 | T24 | 5.91±0.20 | 58.09±4.60 | 0.591±0.208 |
| 37 | Testa | M0 | T0 | 8.26±0.50 | 44.98±1.77 | 0.750±0.119 |
| 38 | Testa | M0 | T12 | 4.99±0.71 | 48.57±4.16 | 0.721±0.141 |
| 39 | Testa | M0 | T24 | 7.14±0.03 | 45.66±2.87 | 0.740±0.116 |
| 40 | Testa | M1 | T0 | 9.33±0.01 | 53.44±2.56 | 0.609±0.092 |
| 41 | Testa | M1 | T12 | 5.97±0.45 | 55.91±1.45 | 0.718±0.136 |
| 42 | Testa | M1 | T24 | 5.54±0.52 | 54.52±4.60 | 0.769±0.186 |
| 43 | Testa | M2 | T0 | 9.74±0.31 | 54.72±1.36 | 0.582±0.082 |
| 44 | Testa | M2 | T12 | 6.12±1.42 | 59.61±2.99 | 0.733±0.115 |
| 45 | Testa | M2 | T24 | 7.14±1.89 | 49.88±4.30 | 0.646±0.041 |
| 46 | Testa | M3 | T0 | 8.92±0.23 | 58.21±2.22 | 0.692±0.096 |
| 47 | Testa | M3 | T12 | 3.92±1.46 | 56.34±4.89 | 0.654±0.096 |
| 48 | Testa | M3 | T24 | 6.57±0.91 | 61.08±5.04 | 0.744±0.147 |
| 49 | Testa | M4 | T0 | 6.17±0.69 | 59.55±1.27 | 0.672±0.037 |
| 50 | Testa | M4 | T12 | 6.63±1.20 | 58.99±0.83 | 0.685±0.102 |
| 51 | Testa | M4 | T24 | 9.23±0.73 | 64.83±4.04 | 0.645±0.246 |
| 52 | Testa | M5 | T0 | 6.98±0.98 | 56.31±2.98 | 0.740±0.183 |
| 53 | Testa | M5 | T12 | 8.61±0.57 | 58.22±2.85 | 0.691±0.107 |
| 54 | Testa | M5 | T24 | 6.49±0.29 | 65.32±3.60 | 0.705±0.128 |
